# Measuring the Impact of Large Language Models on Academic Success and Quality of Life Among Students with Visual Disability: An Assistive Technology Perspective

**DOI:** 10.3390/bioengineering12101056

**Published:** 2025-09-30

**Authors:** Ibrahim A. Elshaer, Sameer M. AlNajdi, Mostafa A. Salem

**Affiliations:** 1 Department of Management, School of Business, King Faisal University, Al-Ahsaa 31982, Saudi Arabia; 2 King Salman Center for Disability Research, Riyadh 11614, Saudi Arabia; 3 Education Technology Department, Faculty of Education and Arts, University of Tabuk, Tabuk 71491, Saudi Arabia; 4 Deanship of Development and Quality Assurance, King Faisal University, Al-Ahsaa 31982, Saudi Arabia

**Keywords:** large language models, ChatGPT, Gemini, assistive technology, visual impairments, disabilities

## Abstract

In the rapid digital era, artificial intelligence (AI) tools have progressively arisen to shape the education environment. In this context, large language models (LLMs) (i.e., ChatGPT vs. 4.0 and Gemini vs. 2.5) have emerged as powerful applications for academic inclusion. This paper investigated how using and trusting LLMs can impact the academic success and quality of life (QoL) of visually impaired university students. Quantitative research was conducted, obtaining data from 385 visually impaired university students through a structured survey design. Partial Least Squares Structural Equation Modelling (PLS-SEM) was implemented to test the study hypotheses. The findings revealed that trust in LLMs can significantly predict LLM usage, which in turn can improve QoL. While LLM usage failed to directly support the academic success of disabled students, but its impact was mediated through QoL, suggesting that enhancements in well-being can contribute to higher academic success. The results highlighted the importance of promoting trust in AI applications, along with developing an accessible, inclusive, and student-centred digital environment. The study offers practical contributions for educators and policymakers, shedding light on the importance of LLM applications for both the QoL and academic success of visually impaired university students.

## 1. Introduction

Artificial intelligence (AI)-based solutions now hold significant potential in terms of overcoming long-standing barriers faced by students with disabilities (particularly those who are blind or visually impaired), such as limited physical assistance and restricted access to educational content and public services [1]. When aligned with universal design principles, AI can support the development of adaptable, user-centred technologies that enhance quality of life and promote active participation in academic, social, and professional contexts [2]. Although many international frameworks advocate disability inclusion, specific guidance on the implementation of AI remains limited. The World Health Organization (WHO), for example, emphasises accessibility in its disability and health guidelines, yet lacks detailed protocols for integrating AI-based solutions (WHO, 2023) [3]. This gap underscores the urgent need for targeted technology policies and inclusive standards that cater to the specific needs of blind and visually impaired learners within the context of digital transformation [4].

The integration of artificial intelligence into accessibility solutions is advancing rapidly. Technologies such as natural language processing (NLP) and computer vision are being deployed in increasingly innovative ways [5]. For example, real-time captioning, sign language interpretation, and intelligent navigation systems significantly enhance communication and mobility for individuals with hearing and visual impairments. However, the deployment of these tools remains inconsistent, often constrained by socioeconomic disparities and geographic inequalities [6]. This underscores the urgent need for standardized accessibility guidelines and user-driven design frameworks that authentically reflect the lived experiences of blind and visually impaired students [7].

Moreover, generative technologies—particularly Large Language Models (LLMs) such as ChatGPT, Gemini, LLaMA, Mixtral, and Claude—are fundamentally reshaping the landscape of digital accessibility [8]. Since the advent of transformer-based architecture, these models have demonstrated exceptional proficiency in generating text, images, audio, and video that are often indistinguishable from human-created content [9]. A comprehensive understanding of both their capabilities and limitations is essential for developing inclusive, ethical applications that effectively serve diverse user populations [10]. However, as AI evolves, it often outpaces regulatory frameworks, resulting in gaps in oversight and accountability [11]. Addressing these challenges requires cross-sector collaboration among developers, regulators, educators, and disability advocates to ensure that AI development remains both responsible and inclusive [6].

With the rise of assistive technologies—including APIs, AI platforms, and LLMs—investigators are revisiting history with a critical lens. Suppose Louis Braille (founder of the Braille system) [12], Valentin Haüy (founder of the first school for blind youth) [13], or Samuel Gridley Howe (founder of the Perkins School for the Blind) [14] had access to today’s AI tools—how might they have reimagined education and social inclusion for visually impaired learners? Similarly, what role would William B. Wait (creator of the New York Point system) [15] have envisioned for AI in advancing access, learning, and empowerment? These questions underscore the transformative potential of artificial intelligence, not merely as a functional tool, but as a catalyst for redefining accessibility in the digital age. The present study explores how LLM-driven technologies can serve as accessible, intelligent tools to support independent learning, academic performance, and well-being among students with visual impairments.

## 2. Literature Review

### 2.1. Large Language Models and Accessibility in Education

The advent of Large Language Models (LLMs) represents a transformative shift in education, particularly in enhancing accessibility for students with diverse learning needs—including those with disabilities, and more specifically, blind and visually impaired learners [16]. Models such as ChatGPT (OpenAI-USA- San Francisco) and Gemini (Google DeepMind-UK-London), trained on vast linguistic datasets, exhibit advanced capabilities in text comprehension, generation, and contextual adaptation [17]. These strengths offer unprecedented opportunities for supporting learners with visual impairments, cognitive differences, and other accessibility challenges [18]. A key advantage of LLMs is their ability to provide real-time, adaptive learning support. They can simplify complex texts for students with dyslexia or cognitive processing difficulties, summarize dense academic content to enhance comprehension, and assist learners through plain-language paraphrasing or translation services [19]. For blind or visually impaired students, LLMs integrated with voice interfaces and screen readers enable natural, interactive engagement, thereby reducing reliance on static or traditional assistive technologies [20]. Their multimodal capabilities—including text-to-speech conversion, alt-text generation for images, and audio descriptions—further democratize access to educational resources [21].

Recent studies underscore the efficacy of LLMs in special education contexts. For instance, learners who use AI-powered tutoring systems show improvements in information retention, faster task completion, and enhanced self-confidence—outcomes attributed to personalised feedback, dynamic scaffolding, and reduced cognitive load, all of which are typically lacking in traditional, one-size-fits-all instructional materials [22,23,24]. These gains stem from personalised feedback, dynamic scaffolding, and reduced cognitive load—advantages often absent in traditional, one-size-fits-all materials [21]. Both Safdar, Samina, and Farrukh [25] and Robinson, Bose, and Cross [26] highlight that LLMs also support educators in designing accessible curricula. These models can generate Universal Design for Learning (UDL)-compliant lesson plans, adapt assessments for diverse learners, and automate the creation of alternative text descriptions or simplified content, thereby alleviating the workload on teachers—especially those without specialized training in accessibility.

Despite their promise, LLMs also present several challenges. These include biases in training data that can reinforce systemic inequities, hallucinations or factual inaccuracies that risk misleading vulnerable learners, and privacy concerns related to handling sensitive educational data [27]. Addressing these risks requires transparent model development, human-in-the-loop oversight, and the inclusive curation of training datasets to ensure fairness and reliability [28]. Ultimately, LLMs are redefining accessibility in education by dynamically adapting content, enabling interactivity, and accommodating a spectrum of learner needs—bridging critical gaps that traditional assistive technologies often cannot [29]. With responsible implementation and ethical oversight, these technologies hold the power to democratize learning and empower all students to reach their full potential [30].

### 2.2. Trust in AI and Assistive Technologies

Artificial intelligence (AI) has become an indispensable part of modern life, playing an increasingly vital role in daily activities, particularly in education for individuals with disabilities, such as blind or visually impaired students. AI has demonstrated significant advancements over traditional solutions, leading to a rapid rise in AI-based educational approaches [31]. However, the widespread adoption of these technologies heavily depends on user trust, as distrust can hinder their acceptance and effectiveness [32]. Trust in AI can be defined as the willingness of individuals to rely on AI systems, accept their suggestions, share tasks, and contribute information [33]. For students with disabilities, AI can only serve as a dependable educational tool if it aligns with their expectations and needs, making trust a critical factor [34]. Trust is established when users can anticipate the system’s behaviour and determine whether it aligns with their goals [35]. Without integrating trust into the development, deployment, and use of AI, its full potential for individuals, organizations, and society will remain unrealized. Thus, it is essential to understand the definition, scope, and role of trust in AI, along with its influencing factors and application-specific requirements [36].

Trust in AI is multidimensional, encompassing perceptions of reliability, transparency, ethical integrity, and responsiveness [37]. Afroogh et al. [38] and Küper and Krämer [39] identified key factors influencing trust in Large Language Models (LLMs), including accuracy, ethical alignment, competence, and transparency. They also highlighted that trust is not solely based on technical performance, but also on the perceived alignment between AI responses and user values, which is a crucial consideration for students with disabilities, who may already face barriers to inclusion and thus demand higher standards of accountability from technology. For visually impaired learners, trust in assistive AI tools directly impacts academic participation and self-efficacy [40]. Studies indicate that students are more likely to adopt tools like screen readers, text-to-speech converters, and AI-generated summaries when outputs are consistently accurate and contextually appropriate [41,42,43]. Conversely, errors, such as mistranslations, misleading paraphrases, or biased content, can erode trust, leading to reduced usage or abandonment of the tool [33,38].

Research in human–computer interaction further supports the dynamic nature of trust, which evolves through positive user experiences [32,37,39]. Geethanjali and Umashankar [44] emphasise that trust in automation can be strengthened through transparency, effective error handling, and the predictability of student needs. In educational contexts, this entails clearly communicating system limitations, allowing users to correct outputs, and ensuring culturally and linguistically sensitive responses [31]. When AI tools incorporate these principles, users are more likely to integrate them into their learning routines and daily lives.

Accordingly, the first research objective (RO1) of this study is to examine the direct effect of trust in LLMs on academic usage, quality of life, and academic success among visually impaired students. This objective informs the following hypotheses:

- H_1_: Trust in LLMs positively predicts LLMs usage.
- H_2_: Trust in LLMs positively predicts quality of life.
- H_3_: Trust in LLMs positively predicts academic success.

### 2.3. Academic Success and Quality of Life with LLM-Supported Learning

In the evolving landscape of inclusive education, Artificial Intelligence (AI)—particularly Large Language Models (LLMs) such as ChatGPT and Gemini—holds transformative potential for enhancing both academic achievement and quality of life for students with disabilities, especially those who are blind or visually impaired [14]. For these learners, educational success extends beyond traditional performance metrics to encompass perceived improvements in comprehension, classroom participation, and conceptual mastery [26]. This holistic perspective is reflected in the construct of academic success and quality of life, which prioritises learner experience and intellectual development alongside, or even above, conventional grade-based assessments [45]. LLM-supported learning tools provide adaptive, accessible, and personalised educational experiences, significantly reducing cognitive, behavioural, and skills barriers [46]. For instance, LLMs can convert visual content into coherent audio formats, summarize complex academic materials, and deliver real-time explanations, all of which are critical for blind or visually impaired students who often encounter unequal access to textual and graphical information [16]. By improving content accessibility, these technologies foster greater engagement in self-directed learning and promote equitable participation in educational settings, thereby contributing to narrowing achievement gaps between visually impaired and sighted learners [19]. Moreover, the integration of LLMs into academic routines has been shown to enhance academic self-efficacy [47]. Visually impaired students benefit from immediate, individualized feedback and tailored support, which helps build confidence in managing academic tasks [48]. This outcome is consistent with the Universal Design for Learning (UDL) principles, which advocates for flexible, inclusive educational environments that accommodate a diverse range of learner needs [49].

Beyond academic performance, LLM use has broader implications for psychosocial well-being. In addition to direct effects, academic usage of LLMs plays a mediating role by translating trust into improved quality of life and academic outcomes. Bach et al. [50] emphasise that when visually impaired students trust and actively engage with technological tools during learning, they gain better access to information and experience greater satisfaction, resulting in enhanced academic performance through empowering educational experiences.

Thus, the second research objective (RO2) is to investigate the mediating role of academic usage in the relationship between trust in LLMs and both quality of life and academic success. This leads to the following hypotheses:

- H_4_: LLM usage positively predicts quality of life.
- H_5_: LLM usage positively predicts academic success.

Michalos [51] define quality of life as the subjective assessment of one’s overall well-being—an outcome positively associated with educational inclusion and access to supportive technologies. Recent studies indicate that assistive and inclusive technologies, particularly those that alleviate everyday frustrations, enhance autonomy, and promote meaningful engagement, can significantly improve the well-being of students with disabilities [52,53,54]. In this regard, LLMs contribute not only by supporting learning but also by reducing dependency on human intermediaries, facilitating smoother academic interactions, and enabling visually impaired students to participate more confidently in both educational and social domains [25,26]. These advantages translate into reduced anxiety, greater motivation, and a heightened sense of belonging within academic communities.

Quality of life also emerges as a crucial mediating factor linking both trust in LLMs and academic usage to academic success. When students experience improved psychological well-being due to active and meaningful engagement with LLMs, their academic performance tends to improve. This suggests that emotional well-being and life satisfaction can indirectly enhance educational outcomes through trusted engagement with LLM tools [47].

Therefore, the third research objective (RO3) is to assess the mediating role of quality of life in the relationship between trust in LLMs, academic usage, and academic success. The corresponding hypotheses are

- H_6_: Quality of life positively predicts academic success.
- H_7_: Trust in LLMs indirectly predicts academic success via quality of life.
- H_8_: Trust in LLMs indirectly predicts quality of life via LLM usage.

Building on global and national initiatives to promote inclusive education—particularly within the context of Saudi Arabia—this study investigates the adoption and use of AI assistive technologies, specifically Large Language Models (LLMs) such as ChatGPT and Gemini, among visually impaired and blind students in Saudi Arabian higher education. While numerous studies have explored the applications of LLMs in the Saudi context, there remains a significant gap in research focusing on their role as catalysts for academic success and quality of life among visually impaired students. This study addresses that gap by examining how these technologies can serve as digital bridges to improve educational outcomes and overall well-being. To explore the underlying factors influencing behavioural intention and actual usage, the study adopts the Unified Theory of Acceptance and Use of Technology (UTAUT) [55], providing evidence-based insights for policymakers, educators, and institutional leaders seeking to implement AI-enabled support systems that promote equity, inclusion, and academic excellence. UTAUT has been extensively investigated in several studies on AI adoption, particularly in the context of assistive technology adoption. While the current paper acknowledges the importance of employing UTAUT as a theoretical background guide, it did not directly use the common factors of UTAUT constructs (i.e., performance expectancy, effort expectancy, social influence, and facilitating conditions). Instead, UTAUT is employed to shed light on the broader classical perspective, linking technology use and trust with student outcomes. Building on this argument, our conceptual framework focuses on two primary constructs—LLMs usage and trust—as the most prominent elements that can influence the academic success and QoL of visually impaired students.

Trust in large language models (LLMs) significantly influences the academic engagement of visually impaired students [16]. High levels of trust in LLMs encourage frequent and effective academic use, enhancing access to learning resources and fostering more inclusive educational experiences [17]. This engagement, in turn, may lead to improved quality of life and academic success by promoting independence, confidence, and equitable learning opportunities [19].

Finally, trust in LLMs is expected to exert indirect effects on academic success through a sequential mediation pathway involving both academic usage and quality of life. Increased trust fosters more consistent and effective LLM usage, which enhances students’ quality of life and, in turn, contributes to improved academic outcomes. This process reflects a compounded mediation mechanism [37].

**Figure 1:**
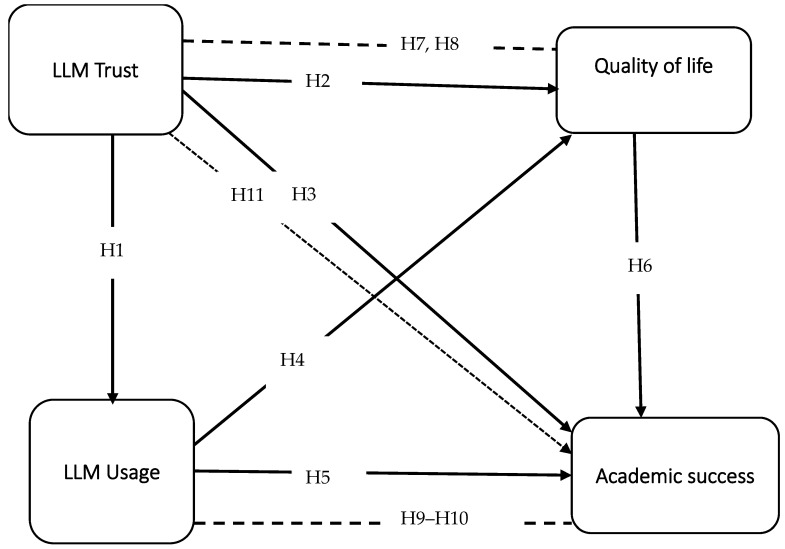
Research framework.

Thus, the fourth research objective (RO4) is to explore the indirect effects of trust in LLMs on academic success through academic usage and quality of life. The final hypothesis ( as seen in Figure 1) is as follows:

- H_9_ Trust in LLMs indirectly predicts academic success via quality of life.
- H_10_ Trust in LLMs indirectly predicts academic success via LLM usage.
- H_11_ Trust in LLMs indirectly predicts academic success via LLM usage and quality of life.

## 3. Methods

### 3.1. The Study Developed Scale

To analytically validate the justified conceptual framework, this paper utilized a structured, self-administered questionnaire as the main data collection tool. The scale and measurement variables were derived from prior, validated scales in the academic literature, ensuring theoretical reliability and methodological accuracy. The questionnaire was organized into three main sections. The opening part provided respondents with a clear and brief explanation of the study’s main aim, along with a consent form developed to confirm their voluntary contribution and ensure ethical criteria. The second part was designed to collect necessary demographic data, including type of student disability, student age group, academic year, and participant gender. The final part addressed the study’s core factors and latent variables. Particularly, academic success was measured by a three-item scale adapted from the study of Owusu-Acheaw and Larson [56]. Although academic success can be employed as a multidimensional concept, concise scale items have been widely used in prior validated research (i.e., [57,58]) to measure students’ perceived academic performance and learning outcomes. In this paper, the sale items were operationalized to reflect the key role of LLMs in improving the educational performance of visually impaired university students. An example item is “The use of large language models (LLMs) has improved my overall learning experience”. Likewise, trust in LLMs was measured via eight items derived from De Duro et al. [59]. These items were carefully reviewed to fit the unique interactional context of LLMs, comprising comfort perceptions, reliability, and willingness. Sample items include “I feel at ease with LLMs, and I can freely share my ideas with them” and “I invest plenty of time developing and improving my prompts to interact with LLMs”. Furthermore, LLM usage was measured using 3-items as suggested by Venkatesh et al. [60]. Sample items include “I intend to use the knowledge and skills I acquired from the LLMs’ in my educational activities. Finally, quality of life was measured by 5 variables of the “Satisfaction with Life Scale” (SWLS). The SWLS was first coined by Diener et al. [61] and developed as a multidimensional item-scale with five variables to reflect individuals’ cognitive judgments concerning their overall life satisfaction. The SWLS is a broadly validated and robust scale designed to measure people’s cognitive assessment of life satisfaction. Its use in this paper enables a reliable evaluation of how visually impaired university students perceive their overall life satisfaction in relation to LLM use. Sampe items include “I am satisfied with my life“ and “The conditions of my life are excellent “. Participants were kindly asked to articulate their level of agreement with several life-related questions using a five-point Likert scale from 1 “strongly disagree” to 5 “strongly agree”. As the employed scale has been extensively validated in prior work, the inclusion offered a strong basis for measurement validity. To emphasise the scale’s face validity, the full design questionnaire was reviewed by eight academics who evaluated its clarity, appropriateness, and the relevance of the included items. Furthermore, a pilot test was conducted with ten students with visual impairments from King Faisal University (KFU). The responses from this preliminary phase showed that the variables were obviously understood, and only minimal language modifications were required. These steps together confirmed that the scale demonstrated good content and face validity.

### 3.2. Population and Sample Size

According to the 2022 Kingdom of Saudi Arabia (KSA) General Population and Housing Census [62], disabilities among residents in KSA encompass various types, including hearing, mobility, cognitive, visual, communication, and self-care challenges. This report indicated that roughly 1.8% of the total KSA population, approximately 648,000 out of 36 million residents, are living with some type of disability. Of these clusters, university students denoted a significant ratio, accounting for around 15.8% (*n* = 102,384). The report also showed that the majority of university students with disabilities are fully enrolled in five main public universities: King Abdulaziz University (1569 disabled students), King Saud University (663 disabled students), Taibah University (523 disabled students), Umm Al-Qura University (381 disabled students), and King Faisal University (330 disabled students). In this paper, these national figures are presented as background context to shed light on the scale of the issue. The actual sampling frame of our paper was independent of these estimates. It was instead based on university students with visual impairments recruited directly from the disability support centres at the universities.

For this paper, the main focus was on containing only university students with visual impairments, with other types of disabilities excluded from the study sample. Respondents were selected based on a convenience sampling technique. To identify the required and adequate sample size, a power analysis was employed by running the G*Power program (version 3.1). The test pattern used the F-test family, pertaining the “Linear multiple regression: Fixed model, R^2^ deviation from zero” option. Selecting a medium size effect (f^2^ = 0.15) with four predictors, and a statistical power of 0.95 with a significance level (α) equal 0.05, the output of the analysis suggested at least 74 responses. To help with the data collection procedure, a team of 40 well-trained enumerators were recruited. These enumerators received instructions regarding the ethical research principles, including how to collect informed consent, safeguard respondents’ confidentiality, and address sensitivity issues related to interactions with university students with disabilities. Orientation workshops were conducted to train enumerators with the study’s purposes and to prepare them to respond to any issues or concerns raised by respondents. Out of the 850 forms that were distributed, 385 were fully answered and fulfilled all validity standards, with a response rate of 45%. The final collected dataset was analysed using Partial Least Squares Structural Equation Modelling (PLS-SEM) to assess both the measurement model and the structural paths model of the unobserved variables. The dataset displayed a reasonably balanced gender distribution, with females encompassing 54% and males 46% of the total participants. Respondents ranged in age from 17 to 24 years old, reflecting a diverse representation of academic levels.

This study relied exclusively on self-reported scale measures of LLM trust and usage, as well as academic success, without obtaining objective system logs or real-time prompts. This methodological selection was primarily driven by several considerations related to privacy, ethical concerns, and accessibility restrictions for visually impaired respondents, many of whom rely on screen readers or adaptive AI technologies that can complicate log-based data collection. Our study recorded the frequency of AI adoption—with 40% of participants reporting occasional LLM usage (e.g., translation or reading help), 44% reporting moderate LLM usage (two–four times per week), and 20% reporting daily LLM usage, with a different academic duties performed (i.e., summarization, translation, grammar checks, information access, and drafting).

### 3.3. Testing Common Method Variance (CMV)

The common method variance (CMV) concern is likely to be presented in social studies research, mainly because the study respondents fill both the dependent and independent variables [63]. As suggested by Williams and Brown [64], this concern can threaten the model’s validity. Following Reio’s [65] recommendation, we conducted some procedural and statistical techniques to alleviate the impact of CMV. First, the questionnaire was developed with several precautionary actions, as suggested by Podsakoff et al. [63], comprising balancing the variables between parts in the questionnaire to minimise instructional impacts; structuring the questions to avoid obvious patterns; and maintaining a suitable questionnaire length. To statistically assess CMV, we run Harman’s single-factor test. The results indicated that the single extracted dimension accounted for 44%, implying that no single dimension explained the majority of the variance, giving strong evidence that CMV concern does not significantly impact our results.

### 3.4. Ethical Approvals

Given the sensitive structure of our research, which included university students with visual impairments, we prioritised the ethical compliance issue. Prior to initiating the data gathering process, we submitted a request to obtain formal institutional approval from the “Institutional Review Board at King Faisal University” (“Ethics Reference: KFU-2025-ETHICS3201, approved 6 October 2024”). This procedure certified that our employed methods were consistent with the institutional criteria and the ethical concerns outlined in the Declaration of Helsinki. We implemented several safeguards to defend participants’ rights: all participation was voluntary in its nature, with no pressure; written informed consent was obtained from each participant; respondents have the right to withdraw at any time without giving any reason; and all data received was anonymized to defend participant identities. Although the collected data contain no personally identifiable information, interested researchers may request access to the obtained data via an official email to the principal investigator.

## 4. Data Analysis and Study Findings

PLS-SEM was run as the primary data analysis method. PLS-SEM is a variance-based technique predominantly suitable for exploratory and predictive research approaches [66]. Distinguished from other covariance-grounded SEM (CB-SEM) [67], PLS-SEM has several advantages: it does not require a normally distributed dataset and can run well with small sample sizes. We conducted the analysis employing SmartPLS program v4, with a bootstrapping procedure and 5000 resamples in the reflective mode [67]. Following the recommendations of Sarstedt et al. [68], the analysis was performed in two subsequent stages. Stage one tested the measurement model’s psychometric properties (Table 1), and stage two tested the structural model for hypotheses confirmation or rejection.

### 4.1. Measurement Model Assessment

We thoroughly evaluated the measurement model by inspecting some main psychometric properties: all employed variables showed high standardized factor loadings (>0.7); the Cronbach’s alpha and composite reliability metrics showed scores that exceeded 0.7; “average variance extracted” (AVE) scores are above the benchmark value of 0.5 [69]. Together, these results support the robustness of the study measures, exhibiting proper internal consistency, scale reliability, and convergent validity. To test discriminant validity, we conducted two complementary methods. First, as suggested by Fornell and Larcker’s [70], we make sure that the square root of each factor’s AVE (presented in Table 2) surpassed all correlations between those factors in the model. This initial evaluation suggested a proper discriminant validity. Additionally, we also assessed the heterotrait–monotrait (HTMT) ratio of correlations [71]. Considered as a more thorough method than the Fornell–Larcker criterion, the HTMT method has potential validity issues when scores surpass the value 0.9. As seen in Table 3, all HTMT scores are below this threshold. Furthermore, the cross-loading values in Table 4 revealed that all variables are highly loaded to their predetermined dimension with no cross-loadings identified. The consistent findings from these two methods offered strong evidence for the discriminant validity of the employed measurement model.

**Figure 2:**
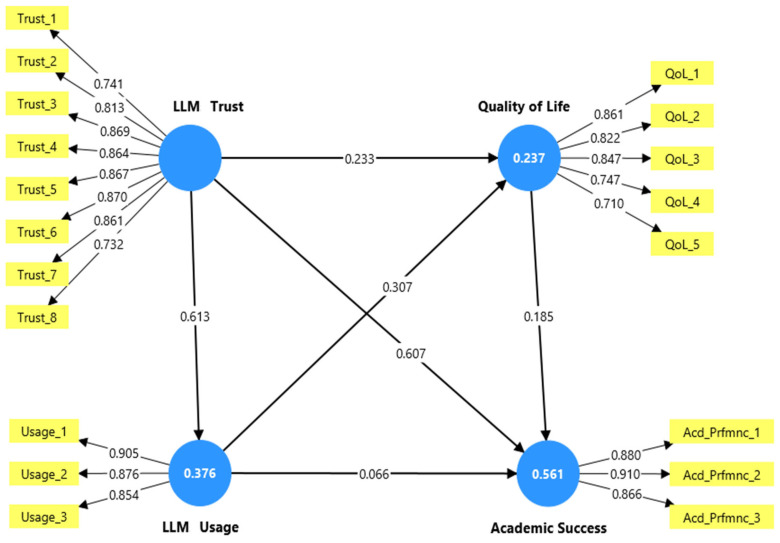
The research model.

The results obtained from PLS-SEM yielded significant insights into the intersection between the tested hypotheses. The bootstrapping findings showed that the three dimensions (LLM trust, LLM usage, and quality of life) can jointly explain 56.1% of the variance in academic success (Figure 2). This high explanatory power implied that the study model can capture most of the main factors impacting academic success.

### 4.2. Structural Model Findings

Before testing the research hypotheses, several Goodness of fit indices were inspected, including SRMR, “coefficient of determination” (R^2^), and “predictive relevance” (Q^2^). As per Hair et al. [67], satisfactory thresholds require SRMR to be less than 0.08, the R^2^ values to be at least 0.10, and Q^2^ scores to surpass zero. The PLS-SEM report indicated that our study model fulfilled these criteria, indicating adequate explanatory and predictive power. Specifically, the SRMR value has an adequate and satisfactory score of 0.07. The academic success endogenous variable has strong predictive power, with R^2^ = 0.561 and Q^2^ = 0.521. This was followed by LLM usage (R^2^ = 0.376, Q^2^ = 0.368) and QoL (R^2^ = 0.237, Q^2^ = 0.167), each displaying adequate levels of the variance explained and predictive power. Finally, to ensure that multicollinearity does not affect model validity, Variance Inflation Factor (VIF) values were inspected, as they should remain under a value of 5, as suggested by [66]. As seen in Table 1, all values are below 5, indicating that no multicollinearity issues existed in our model.

After confirming the reliability and validity of both the measurement model and structural model, hypotheses testing process can proceed. As presented in Table 4, the bootstrapped output of the tested study model revealed that trust in large language models (ChatGPT/Gemini) has a positive and significant impact on LLM usage (β = 0.613, t = 10.895, *p* < 0.001), QoL (β = 0.233, t = 3.745, *p* < 0.001), and academic success (β = 0.607, t = 14.001, *p* < 0.001). Accordingly, H1, H2, and H3 were supported. Furthermore, the PLS-SEM results have introduced evidence that LLM usage had a positive and significant impact on QoL (β = 0.307, t = 4.893, *p* < 0.001), supporting H5. However, interestingly, LLM usage did not directly lead to academic success (β = 0.066, t = 1.652, *p* = 0.099), rejecting H5. Additionally, QOL was found to have a significant influence on academic success (β = 0.185, t = 5.533, *p* <0.001), supporting H6.

The PLS-SEM output also revealed some evidence regarding the specific indirect effects. Trust in LLMs slightly but successfully impacted academic success through QoL (β = 0.043, t = 2.713, *p* < 0.01), supporting H7. However, trust in LLMs failed to significantly impact academic success through LLM usage (β = 0.040, t = 1.499, *p* = 0.134), rejecting H8. Additionally, the specific indirect effects in PLS-SEM revealed that LLM usage has an indirect significant impact on academic success through QoL (β = 0.057, t = 3.496, *p* < 0.001), supporting H9. Similarly, trust in LLMs was found to have an indirect significant impact on QoL through LLM usage (β = 0.188, t = 4.554, *p* ≤ 0.001), supporting H10. Finally, trust in LLMs was found to have a significant indirect impact on academic success through QoL and LLM usage (β = 0.35, t = 3.392, *p* ≤ 0.01), supporting H11, as seen in Table 5.

## 5. Discussion

This paper tested how trust in large language models (LLMs), more specifically ChatGPT and Gemini, foster academic success and affect QoL among university students with visual impairments in KSA. The results revealed that trust in LLMs among visually impaired students in KSA can significantly predict the usage of these AI technologies, as well as their QoL and academic success. These findings provide empirical evidence that highlights the key role of trust as an underlying mechanism for the successful implementation of AI-driven assistive tools in an educational context. The significant influence of LLM trust on usage is consistent with previous research that highlights the key role of trust in user adoption of AI technologies, specifically for underserved or vulnerable populations [50,72]. For students with visual impairments, who regularly depend deeply on AI assistive technology for both academic activities and their personal lives, trust acts as a doorkeeper to engagement. If AI assistive technology is perceived as user-friendly, non-discriminatory, reliable, and easily accessible, people are more likely to integrate it into their daily life routines [73].

Furthermore, the positive impact of LLMs’ trust on QoL resonates with previous literature that has connected digital attachment with well-being among people with various types of disabilities. When AI-assistive technologies, such as ChatGPT or Gemini, can offer timely, understandable, and context-sensitive data, they can minimise barriers to independent existing and external communication, thus enhancing social engagement and psychological well-being [74,75]. Furthermore, the significant link between LLMs trust and academic success suggests that university students who depend on and trust in these AI tools may obtain a performance advantage. LLMs provide adaptive and adequate language support, personalised descriptions, and timely academic assistance for students who encounter accessibility limitations [76]. These results align with recent research that has shown AI-based assistive technologies can foster cognitive access, learning independence, and self-efficacy among students with disabilities [77,78].

Furthermore, the results showed that while LLM usage had a positive and significant influence on students’ QoL, it did not show a direct significant impact on academic success. Surprisingly, the results also confirmed that QoL itself can significantly predict academic success, highlighting a potential indirect relationship. The positive relationship between LLM usage and QoL is aligned with recent research indicating that AI-derived tools can play a meaningful role in enhancing the daily experiences of people with disabilities [73,75]. For visually impaired university students, LLMs serve not only as academic tools but also as life-improving technologies that promote broader social and psychological well-being [74]. Conversely, the absence of a direct link between LLM usage and academic success warrants a deeper investigation. While previous research has suggested that AI technologies may foster learning efficiency and understanding [76], our results indicate that simply using LLMs alone is not sufficient to secure improved academic success. This finding may be explained by the way university students engage with these tools, perhaps using them more for daily information, internet browsing, or emotional support rather than for academic tasks [78]. Moreover, some contextual factors, such as AI literacy, the understanding of rapid engineering, and the capability to assess LLM-generated outcomes, may impact whether LLM usage can be translated into academic achievements. University students who lack the essential skills to structure effective enquiries or who accept AI replies uncritically might fail in leveraging the full academic potential of these AI technologies. Future research can further test moderating elements, such as digital literacy, type of LLM, and different usage types, as well as contextual support, to identify the conditions under which LLM usage can significantly impact academic success.

Notably, the significant positive impact of QoL on academic success highlighted the solid connections between well-being and educational success. As supported by previous research, students who have a higher level of social connection, emotional support, and self-efficacy are in a better situation to excel in their academic life [77]. For visually impaired students, this suggested that AI technologies, by enhancing QoL via reducing other dependencies, encouraging confidence, or fostering social interaction, may indirectly improve academic outcomes, even if their direct academic usefulness is restricted.

Inspection of the specific indirect effects in the PLS-SEM report revealed a set of mediating relationships, highlighting that LLM trust alone may not directly improve academic success, but can indirectly influence academic success through enhanced well-being and engagement with AI tools. Firstly, the PLS-SEM results demonstrated that trust in LLMs significantly impacted academic success via QoL. This suggests that when university students trust these AI technologies, it may foster their overall satisfaction with life through reduced stress, increased self-independence, and a high level of confidence in finalising academic tasks, which may consequently contribute to better academic performance. These results are aligned with prior studies that suggested that well-being can play a key mediating role in academic success, particularly for students with visual disabilities [75]. Nevertheless, trust in LLMs failed to significantly impact academic success through the use of LLMs alone. These findings are both revealing and informative. They imply that simply employing LLMs—even if university students trust them—did not necessarily translated into academic advantages. This may be because of the variation in how university students use these technologies—some might use them for regular inquiries or emotional support rather than to directly accomplish academic duties [78]. The model also revealed that LLM usage positively influences academic success indirectly through QoL. This highlighted an influential relationship: when LLMs are employed in ways that improve students’ daily operations and overall emotional well-being, the subsequent improvements in QoL might fuel a more attentive, motivated, and empowered learning process. These findings align with earlier evidence suggesting that digital accessibility technologies may contribute to high psychological readiness and increased cognitive engagement, which are key drivers of academic success [73,74].

Additionally, substantial indirect impacts were found from LLM trust to QoL via LLM usage. This emphasised the key role of trust as a precursor to successful technological commitment. University students who view LLMs as trustworthy are more likely to adopt them into their daily life routine, fostering convenience and self-independence that together contribute to a higher level of QoL [50,77]. Finally, LLM trust was found to have an indirect impact on academic success via both QoL and LLM usage. These multi-pathway mediation effects revealed that trust can play a grounded role not by immediately fostering academic advantages but by reshaping university students’ interaction with assistive AI technologies, enhancing QoL, and generating a more helpful learning context. These results support calls in the literature for a more holistic, student-focused approach to AI adoption in the educational context [73,76].

## 6. Conclusions

This paper investigated the influence of large language models (LLMs), especially ChatGPT and Gemini, on the academic success and QoL of students with visual impairments in Saudi Arabian higher education institutions. Based on PLS-SEM data analysis techniques, the study results revealed that LLM trust can significantly predict LLM usage, which consequently contributed to improved QoL. While LLM usage failed to generate higher academic performance directly, its positive impact on QoL indirectly encouraged academic success. These results highlight nuanced intersections in which academic achievements can be understood as part of a broader ecosystem of digital trust, emotional well-being, and comprehensive technology usage. Crucially, the paper confirmed that LLMs can act as a meaningful technology for educational equity when LLM usage aligns with the requirements and lived experiences of university students with disabilities. Trust and accessibility continued to be central to their efficiency, especially for visually impaired students who depend on such technologies not only for information purposes but also for the independent learning process and social interaction.

The study revealed several implications for university leaders and policymakers. Universities must adopt a more holistic approach to digital technology inclusion by not only offering access to LLM tools but also providing a supportive context that fosters user trust and enhances digital literacy. Training workshops focused on ethical usage, academic reliability, and LLM task-specific implementations should be incorporated into disability support services practices. Furthermore, guidelines should be updated to clearly incorporate LLMs as recognised assistive technologies within the university context for inclusive education. The study also provided some crucial implications for scholars: it highlighted the significance of investigating assistive AI technology not only from a performance point of view but also in terms of its impact on well-being. Future research papers should consider a longitudinal research design to detect the evolving intersections between LLM usage and university student improvements over time. Moreover, expanding this study to other disability types (beyond visual impairments) and cultural contexts (beyond university students) can provide a more inclusive understanding of the transformative potential of AI technologies. While several statistical and procedural approaches were implemented to deal with Common Method Bias (CMB), future research could conduct more advanced statistical and procedural techniques—such as temporal separation of measurement, unmeasured latent method factor analysis, or scale separation principles—to more thoroughly address possible CMB.

Furthermore, a key limitation of this paper is that it relied on self-reported measures of LLM trust and usage, as well as academic achievement, without obtaining objective measures such as system logs or actual-time prompts. Future research papers are consequently encouraged to use mixed-method approaches, obtaining both subjective and objective measures to create a richer understanding of how visually impaired students engage with LLMs in the academic environment. One more promising avenue for future studies lies in assessing the demographic subgroup analyses to better understand how demographic characteristics (i.e., gender type, age level, or educational discipline) may impact LLM trust and usage, academic success, and QoL. While this study obtained demographic data, multigroup analysis and measurement invariance were beyond the main scope of this study. Prior studies (e.g., Thakur et al. [79]; Fosch-Villaronga et al. [80]) have argued that demographic diversity can influence technology acceptance and user outcomes, highlighting the importance of incorporating these demographic differences into future research. Finally, although this paper can offer important implications, its results must be understood within the educational and cultural context of SA. These factors could impact both the level of LLM trust and the potential outcomes linked to their usage. As such, the generalizability of the results to other educational or cultural contexts should be approached with caution.

## Figures and Tables

**Table 1:** Factor loadings and scale psychometric properties.

	FL	α	C.R.	AVE	VIF
Large Language Model Trust		0.934	0.935	0.687	
Trust_1	0.741				2.069
Trust_2	0.813				2.472
Trust_3	0.869				2.163
Trust_4	0.864				2.644
Trust_5	0.867				3.094
Trust_6	0.870				3.076
Trust_7	0.861				3.128
Trust_8	0.732				1.909
Large Language Model Usage		0.852	0.858	0.772	
Usage_1	0.905				2.350
Usage_2	0.876				2.115
Usage_3	0.854				1.936
Quality of life		0.859	0.879	0.640	
QoL_1	0.861				2.403
QoL_2	0.822				2.236
QoL_3	0.847				2.217
QoL_4	0.747				2.036
QoL_5	0.710				1.877
Academic success		0.863	0.868	0.784	
Acd_Prfmnc_1	0.880				2.034
Acd_Prfmnc_2	0.910				2.783
Acd_Prfmnc_3	0.866				2.209

Note. FL = Factor Loading; α = Cronbach’s Alpha; C.R. = Composite Reliability; AVE = Average Variance Extracted; VIF = Variance Inflation Factor. Recommended thresholds: FL > 0.70, α > 0.70, CR > 0.70, AVE > 0.50, VIF < 5.0.

**Table 2:** “Fornell and Larcker”.

	Academic Success	LLM Trust	LLM Usage	Quality of Life
Academic Success	0.886			
LLM Trust	0.725	0.829		
LLM Usage	0.521	0.613	0.879	
Quality of Life	0.470	0.422	0.450	0.800

**Table 3:** “Heterotrait–monotrait ratio” (HTMT)—Matrix.

	Academic Success	LLM Trust	LLM Usage	Quality of Life
Academic Success				
LLM Trust	0.803			
LLM Usage	0.602	0.683		
Quality of Life	0.532	0.464	0.515	

**Table 4:** Factor cross-loadings.

	Academic Success	LLM Trust	LLM Usage	Quality of Life
Acd_Prfmnc_1	0.880	0.708	0.494	0.440
Acd_Prfmnc_2	0.910	0.618	0.426	0.397
Acd_Prfmnc_3	0.866	0.589	0.458	0.408
QoL_1	0.426	0.395	0.451	0.861
QoL_2	0.376	0.311	0.330	0.822
QoL_3	0.461	0.375	0.399	0.847
QoL_4	0.265	0.281	0.324	0.747
QoL_5	0.309	0.302	0.262	0.710
Trust_1	0.615	0.741	0.554	0.329
Trust_2	0.629	0.813	0.576	0.349
Trust_3	0.548	0.869	0.549	0.324
Trust_4	0.601	0.864	0.443	0.364
Trust_5	0.607	0.867	0.508	0.409
Trust_6	0.557	0.870	0.566	0.367
Trust_7	0.605	0.861	0.481	0.335
Trust_8	0.639	0.732	0.359	0.308
Usage_1	0.512	0.572	0.905	0.423
Usage_2	0.462	0.526	0.876	0.379
Usage_3	0.391	0.515	0.854	0.382

**Table 5:** Path coefficient and related *t* and *p* values.

Hypotheses	β	T	*p*	Results
LLM Trust -> LLM Usage	0.613	10.895	0.000	H1: Supported
LLM Trust -> Quality of Life	0.233	3.745	0.000	H2: Supported
LLM Trust -> Academic Success	0.607	14.001	0.000	H3: Supported
LLM Usage -> Quality of Life	0.307	4.893	0.000	H4: Supported
LLM Usage -> Academic Success	0.066	1.652	0.099	H5: Rejected
Quality of Life -> Academic Success	0.185	5.535	0.000	H6: Supported
**Specific indirect effects**				
LLM Trust -> Quality of Life -> Academic Success	0.043	2.713	0.007	H7: Supported
LLM Trust -> LLM Usage -> Academic Success	0.040	1.499	0.134	H8: Rejected
LLM Usage -> Quality of Life -> Academic Success	0.057	3.496	0.000	H9: Supported
LLM Trust -> LLM Usage -> Quality of Life	0.188	4.554	0.000	H10: Supported
LLM Trust -> LLM Usage -> Quality of Life -> Academic Success	0.035	3.392	0.001	H11: Supported

## Data Availability

The data presented in this study are available on request from the corresponding author due to its privacy.
